# Prospective implementation of AI-assisted screen reading to improve early detection of breast cancer

**DOI:** 10.1038/s41591-023-02625-9

**Published:** 2023-11-16

**Authors:** Annie Y. Ng, Cary J. G. Oberije, Éva Ambrózay, Endre Szabó, Orsolya Serfőző, Edit Karpati, Georgia Fox, Ben Glocker, Elizabeth A. Morris, Gábor Forrai, Peter D. Kecskemethy

**Affiliations:** 1 Kheiron Medical Technologies, London, UK; 2 MaMMa Egészségügyi Zrt., Budapest, Hungary; 3 Department of Computing, Imperial College London, London, UK; 4 University of California, Davis, Davis, CA USA; 5 Duna Medical Center, Budapest, Hungary

**Keywords:** Breast cancer, Population screening, Radiography, Diagnosis

## Abstract

Artificial intelligence (AI) has the potential to improve breast cancer screening; however, prospective evidence of the safe implementation of AI into real clinical practice is limited. A commercially available AI system was implemented as an additional reader to standard double reading to flag cases for further arbitration review among screened women. Performance was assessed prospectively in three phases: a single-center pilot rollout, a wider multicenter pilot rollout and a full live rollout. The results showed that, compared to double reading, implementing the AI-assisted additional-reader process could achieve 0.7–1.6 additional cancer detection per 1,000 cases, with 0.16–0.30% additional recalls, 0–0.23% unnecessary recalls and a 0.1–1.9% increase in positive predictive value (PPV) after 7–11% additional human reads of AI-flagged cases (equating to 4–6% additional overall reading workload). The majority of cancerous cases detected by the AI-assisted additional-reader process were invasive (83.3%) and small-sized (≤10 mm, 47.0%). This evaluation suggests that using AI as an additional reader can improve the early detection of breast cancer with relevant prognostic features, with minimal to no unnecessary recalls. Although the AI-assisted additional-reader workflow requires additional reads, the higher PPV suggests that it can increase screening effectiveness.

## Abstract (web summary)

In a phased prospective rollout, the implementation of AI as an additional reader for mammography screening improved the real-world early detection of breast cancer compared to standard double reading involving two independent radiologists.

## Main

Breast cancer screening detects cancer at earlier stages^1^, leading to a meaningful reduction in breast cancer mortality^2^. Moreover, early detection can lead to less aggressive treatments, reducing treatment toxicity. Although breast screening reduces overall mortality, it has limitations that result in failure to detect cancer in a considerable number of screened individuals. In these cases, cancer may be found later between screening rounds (interval cancer)^3^ or at the next screening round^4^. Reported estimates for the rate of interval cancer detection vary widely between countries and screening programs with varying screening intervals, ranging from 0.7 to 4.9 per 1,000 screened women^3^. Among them, the proportion of cancer cases that could be detected retrospectively at previous rounds is estimated to be 22%^4^. In the past, computer-aided detection (CAD) systems were developed to improve cancer detection. However, the benefits of CAD found in experimental studies did not translate into real-world clinical benefits. The use of CAD resulted in increased recalls, more time needed to assess screens and more biopsies without improving cancer detection, ultimately conferring no screening benefit^5^.

Modern artificial intelligence (AI) based on deep learning is a different technology from past CAD systems and has demonstrated higher potential in supporting the quality of screening services and reducing workload, depending on its workflow integration^6–10^. AI has the highest performance risk for cases with less common characteristics; thus, it requires assessment in large-scale studies. As retrospective studies make large-scale evaluations possible, they are crucial to validate the safety and effectiveness of AI before prospective use. However, retrospective results can be expected to translate to real clinical practice only when appropriate study methods are used to ensure that the analyzed data are representative of what AI would process in real-world deployments. Otherwise, the usefulness of AI in clinical practice is not guaranteed^4,11,12^. Prospective evaluations are needed to assess the real-world performance of AI integrated into live clinical workflows; however, these have been limited to date^13^.

This service evaluation presents results from using a commercially available AI system, Mia (Kheiron Medical Technologies), configured with regulatory-cleared predetermined sensitivity and specificity operating points in pilot implementations and live use in daily practice. The performance and generalizability of the AI system used were previously confirmed in a large-scale retrospective AI generalizability study^8,9,14^. The current analysis used prospectively collected postmarket real-world data to assess the effectiveness of the AI system as an additional component to standard screening procedures and a quality-control safety net in the AI-assisted additional-reader workflow to support early cancer detection.

## Results

**Fig. 1:**
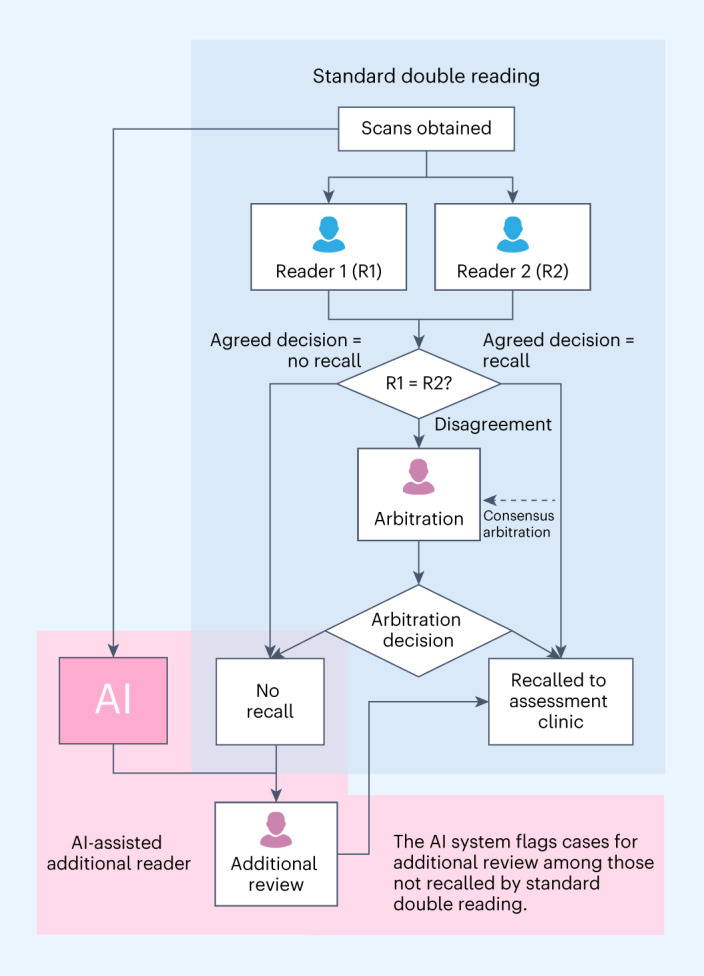
AI as an additional reader. The AI-assisted additional-reader workflow uses a standard double-reading process complemented by image assessment by AI. If double reading results in a ‘no recall’ decision but the AI system flags the case, the screen is assessed by an additional human arbitrator.

A three-phase approach was used to implement the AI system in an AI-assisted additional-reader workflow at four sites of MaMMa Egészségügyi Zrt. (MaMMa Klinika), a breast cancer screening institution that serves urban and rural populations in Hungary. The institution implements a 2-year screening interval and invites women aged 45–65 years to undergo screening. All institution sites also offer opportunistic screening, in which women who are not invited to screening but choose to participate are screened. These women undergo the same procedure as those participating in the population screening program. At the institution sites, full-field digital mammography images were obtained using the IMS Giotto Image 3DL and IMS Giotto Class systems, following the standard operating procedures at the four sites. All sites follow the standard double-reading workflow (with strictly no AI involvement) in which two radiologists review every case. When discordance arises, an arbitrator makes the decision to either recall or not recall a woman for further assessment. In the implemented AI-assisted additional-reader workflow, the AI system flagged cases for additional review among those classified by double reading as ‘no recall’. These positive discordant cases (that is, cases that AI flagged as ‘positive’ and human readers marked as ‘negative’) were additionally reviewed by a human arbitrator (additional arbitrator) to possibly recall additional cases and detect more cancerous cases at an early stage (Fig. 1). The additional arbitrator was provided with images containing AI-generated regions of interest highlighting areas suggestive of malignancy for their review.

The implementation of the AI system consisted of three phases to ensure the safe deployment of the AI-assisted additional-reader process into live use. The first phase aimed to demonstrate the clinical benefit of the AI-assisted additional-reader process in a limited pilot rollout in which only one senior radiologist reviewed the AI-flagged cases from a single site, with the original screening date between April 6 and September 28, 2021 inclusive. The second phase was launched as an extended multicenter pilot involving a wider rollout of the AI-assisted additional-reader process across four sites (including the initial pilot site) and three additional arbitrators (including the additional arbitrator from the first phase). In the second phase, the readers independently reviewed every case flagged by AI from April 6 through December 21, 2021, at the initial pilot site and from April 6 through June 30, 2021, at each of the other three sites. One of the additional arbitrators made the final decision on which cases to recall additionally based on the opinions of all three readers. The extended pilot also aimed to provide a training period for the three additional arbitrators before live use began.

Finally, the third phase involved a full live rollout of the AI system as an official addition to the standard of care across the four sites from July 4, 2022. In this phase, the three additional arbitrators independently made recall decisions. The live rollout is ongoing, and the results presented here cover cases through January 31, 2023. Results were also simulated with a predetermined higher-specificity operating point to inform the sites on how the AI-assisted additional-reader process may be further optimized to suit their needs. The summary details of the dataset periods are provided in Table 1. In live use, each AI-flagged case was independently reviewed by one of the three additional arbitrators who made the final recall decision on each case they reviewed. During the two pilot phases, additional recalls based on additional arbitration reviews were done after the screening participants had been informed of the double-reading decision. In the third phase involving implementation into daily practice, the screening participants were informed after the decision was finalized based on the additional arbitration reviews. All readers had specialist training and ≥14 years of screening mammography experience, with non-additional arbitrators reading approximately 12,000 screens per year and additional arbitrators reading 25,000 screens per year on average.

**Table 1:** Overview of screens per phase per site

Site	First month	Final month	Vendor	Equipment model	No. of available double-read screens	No. of processed screens	Percentage
Phase 1, initial pilot (1 site, 1 additional arbitrator, additional arbitration cases were single read)							
Site 1	April 2021	September 2021	IMS	Giotto Class	3,817	3,746	98.1%
Phase 2, extended pilot (4 sites, 3 additional arbitrators, all additional arbitration cases were read by each additional arbitrator)							
Site 1	April 2021	December 2021	IMS	Giotto Class	5,859	5,758	98.3%
Site 2	April 2021	June 2021	IMS	Giotto Class	1,187	1,172	98.7%
Site 3	April 2021	June 2021	IMS	Giotto Image 3DL	918	911	99.2%
Site 4	April 2021	June 2021	IMS	Giotto Image 3DL	1,302	1,271	97.6%
Total					9,266	9,112	98.3%
Phase 3, live use in standard clinical practice (4 sites, 3 additional arbitrators, additional arbitration cases were single read)							
Site 1	July 2022	January 2023	IMS	Giotto Class	4,818	4,711	97.8%
Site 2	July 2022	January 2023	IMS	Giotto Class	4,605	4,537	98.5%
Site 3	July 2022	January 2023	IMS	Giotto Image 3DL	2,925	2,903	99.2%
Site 4	July 2022	January 2023	IMS	Giotto Image 3DL	3,908	3,802	97.3%
Total					16,256	15,953	98.1%

### Patient characteristics

Table 2 shows the characteristics of participants in each phase. The initial pilot included 3,746 women with an average age of 58.2 (s.d. 11.0) years. Among them, 126 (3.4%) reported a family history of cancer and 479 (12.7%) had a Tabár parenchymal pattern classification of 4 or 5, correlating with high density. In the extended pilot (*n* = 9,112), the mean age was also 58.2 (s.d. 10.7) years. Tabár classification 4 or 5 was identified in 1,094 women (12.0%), and 274 women (3.0%) reported a family history of cancer. Finally, in the live-use phase, 15,953 women were included. The mean age was 58.6 (s.d. 10.5) years, with 615 women (3.9%) having reported a family history of cancer and 1,733 women (10.8%) having a Tabár classification of 4 or 5.

**Table 2:** Participant characteristics per phase

Variable	Initial pilot (*n* = 3,746)	Extended pilot (*n* = 9,112)	Live use (*n* = 15,953)
Age (continuous, years), mean (s.d.)	58.2 (11.0)	58.2 (10.7)	58.6 (10.5)
Age group, *n* (%)			
≤35 years	0 (0.0%)	0 (0.0%)	0 (0.0%)
36–45 years	518 (13.8%)	1,149 (12.6%)	1,583 (9.9%)
46–55 years	1,218 (32.5%)	2,998 (32.9%)	5,420 (34.0%)
56–65 years	940 (25.1%)	2,493 (27.4%)	4,699 (29.5%)
66–75 years	806 (21.5%)	1,902 (20.9%)	3,196 (20.0%)
>75 years	264 (7.0%)	570 (6.3%)	1,055 (6.6%)
Family history^a^, *n* (%)			
No	3,620 (96.6%)	8,838 (97.0%)	15,338 (96.1%)
Yes	126 (3.4%)	274 (3.0%)	615 (3.9%)
Tábar classification of parenchymal patterns^b^, *n* (%)			
1	1,506 (40.2%)	3,950 (43.3%)	7,468 (46.8%)
2	729 (19.5%)	1,697 (18.6%)	2,921 (18.3%)
3	336 (9.0%)	679 (7.5%)	465 (2.9%)
4	365 (9.7%)	848 (9.3%)	1,423 (8.9%)
5	114 (3.0%)	246 (2.7%)	310 (1.9%)
Missing	696 (18.6%)	1,692 (18.6%)	3,366 (21.1%)

^a^Family history of cancer = ‘yes’ if at least two first-degree female family members have been diagnosed with breast cancer.

^b^A Tabár classification^17^ of 4 or 5 correlating with high density (BI-RADS (breast imaging and reporting data system) breast density class C or D).

### Screening performance of the AI-assisted additional-reader workflow

Across the three phases, the implementation of the AI-assisted additional-reader workflow resulted in 24 more cancer cases detected (7% relative increase in cancer detection rate (CDR)) and 70 more women recalled (0.28% increase in absolute recall rate), at a positive predictive value (PPV) for screening of 20.0% (3% relative increase) (Table 3). The initial pilot, extended pilot and live-use assessments included 3,746 of 3,817 (98.1%), 9,112 of 9,266 (98.3%) and 15,953 of 16,256 (98.1%) double-read cases that the AI could process, respectively (Table 1). Table 3 shows the outcome metrics for each phase and reports the results of the McNemar test for sensitivity and CDR. In summary, standard double reading resulted in recall rates of 6.7% (initial pilot), 7.0% (extended pilot) and 7.7% (live use) and CDRs of 12.8 per 1,000 cases (initial pilot), 13.8 per 1,000 cases (extended pilot) and 14.9 per 1,000 cases (live use). For the initial and extended pilots, AI flagged for review 10.6% (396/3,746) and 11.2% (1,024/9,112) of cases, respectively. Before launching the AI system into live use, its decision threshold was adjusted to a more specific predetermined operating point to accommodate the site’s workload capacity, resulting in a smaller proportion of cases (7.4%, 1,186/15,953) flagged for additional review in live use. The additional arbitration reviews resulted in six (initial pilot), 22 (extended pilot) and 48 (live use) additional recalled cases, increasing the recall rate by 0.16% (initial pilot), 0.23% (extended pilot) and 0.25% (live use), respectively. From the additional recalls, six (initial pilot), 13 (extended pilot) and 11 (live use) additional cancer cases were found, increasing the CDR by 1.6 per 1,000 cases (a 13% relative increase), 1.4 per 1,000 cases (a 10% relative increase) and 0.7 per 1,000 cases (a 5% relative increase) for the initial pilot, extended pilot and live-use phases, respectively (all statistically significant with *P* < 0.05) (Table 3). Of the additional cancer cases, four (66.7%) in the initial pilot, ten (76.9%) in the extended pilot and five (45.5%) in the live-use phase were confirmed to be invasive. In addition, one case (16.7%) in the initial pilot, one case (7.7%) in the extended pilot and two cases (18.2%) in live use were in situ cancer. Meanwhile, one case (16.7%) in the initial pilot, two cases (15.4%) in the extended pilot and four cases (36.4%) in live use had missing invasiveness information. Of the additional cancer cases found with available data on either pathological or radiological tumor size, 50.0% (two of four) in the initial pilot, 40% (four of ten) in the extended pilot and 57.1% (four of seven) in live use were ≤10 mm. Overall, the screening performance of double reading plus the AI-assisted additional-reader workflow resulted in recall rates of 6.8% (initial pilot), 7.3% (extended pilot) and 8.0% (live use); arbitration rates of 13.6% (initial pilot), 14.2% (extended pilot) and 10.8% (live use); and CDRs of 14.4 per 1,000 cases (initial pilot), 15.3 per 1,000 cases (extended pilot) and 15.6 per 1,000 cases (live use).

**Table 3:** Outcome metrics for standard double reading versus double reading plus the AI-assisted additional-reader workflow

Variable	Double reading		Double reading plus the AI-assisted additional-reader workflow		Difference
	Num/Denom	Value (95% CI)	Num/Denom	Value (95% CI)	
Results of phase 1, pilot rollout (1 site, 1 additional arbitrator, additional arbitration cases were single read), *n* = 3,746 screens					
CDR (per 1,000 cases)	48/3,746	12.8 (9.7–16.9)	54/3,746	14.4 (11.1–18.8)	1.6^a^
RR (%)	250/3,746	6.7 (5.9–7.5)	256/3,746	6.8 (6.1–7.7)	0.2
Sen (%)	48/58	82.8 (71.7–90.4)	54/58	93.1 (83.6–97.3)	10.3^a^
Spec (%)	3,486/3,688	94.5 (93.7–95.2)	3,486/3,688	94.5 (93.7–95.2)	0.0
PPV (%)	48/250	19.2 (14.8–24.5)	54/256	21.1 (16.5–26.5)	1.9
Arbitration rate (%)	114/3,746	3.0 (2.5–3.6)	510/3,746	13.6 (12.6–14.8)	10.6
Positive discordance rate (%)	–	–	396/3,746	10.6 (9.6–11.6)	–
RR of additional arbitration (%)	–	–	6/396	1.5 (0.7–3.3)	–
PPV of additional arbitration (%)	–	–	6/6	100 (61.0–100)	–
Results of phase 2, extended pilot (4 sites, 3 additional arbitrators, all additional arbitration cases were read by each additional reader), *n* = 9,112 screens					
CDR (per 1,000 cases)	126/9,112	13.8 (11.6–16.4)	139/9,112	15.3 (12.9–18.0)	1.4^a^
RR (%)	639/9,112	7.0 (6.5–7.6)	661/9,112	7.3 (6.7–7.8)	0.2
Sen (%)	126/145	86.9 (80.4–91.4)	139/145	95.9 (91.3–98.1)	9.0^a^
Spec (%)	8,454/8,967	94.3 (93.8–94.7)	8,445/8,967	94.2 (93.7–94.6)	−0.1
PPV (%)	126/639	19.7 (16.8–23.0)	139/661	21.0 (18.1–24.3)	1.3
Arbitration rate (%)	270/9,112	3.0 (2.6–3.3)	1,294/9,112	14.2 (13.5–14.9)	11.2
Positive discordance rate (%)	–	–	1,024/9,112	11.2 (10.6–11.9)	–
RR of additional arbitration (%)	–	–	22/1,024	2.1 (1.4–3.2)	–
PPV of additional arbitration (%)	–	–	13/22	59.1 (38.7–76.7)	–
Results of phase 3, live use in standard clinical practice (4 sites, 3 additional arbitrators, additional arbitration cases were single read), *n* = 15,953 screens					
CDR (per 1,000 cases)	238/15,953	14.9 (13.2–16.9)	249/15,953	15.6 (13.8–17.7)	0.7^a^
RR (%)	1,228/15,953	7.7 (7.3–8.1)	1,276/15,953	8.0 (7.6–8.4)	0.3
Sen (%)	238/253	94.1 (90.4–96.4)	249/253	98.4 (96.0–99.4)	4.3^a^
Spec (%)	14,710/15,700	93.7 (93.3–94.1)	14,673/15,700	93.5 (93.1–93.8)	−0.2
PPV (%)	238/1,228	19.4 (17.3–21.7)	249/1,276	19.5 (17.4–21.8)	0.1
Arbitration rate (%)	529/15,953	3.3 (3.0–3.6)	1,715/15,953	10.8 (10.3–11.2)	7.4
Positive discordance rate (%)	–	–	1,186/15,953	7.4 (7.0–7.9)	–
RR of additional arbitration (%)	–	–	48/1,186	4.0 (3.1–5.3)	–
PPV of additional arbitration (%)	–	–	11/48	22.9 (13.3–36.5)	–

Num, numerator; Denom, denominator; CI, confidence interval; Sen, sensitivity; Spec, specificity; RR, recall rate; see metric definitions in Methods.

^a^The two-sided McNemar test to assess CDR and Sen differences between double reading and double reading plus the AI-assisted additional-reader workflow resulted in *P* values of 0.0031, 0.0002 and 0.001 for phases 1, 2 and 3, respectively. The McNemar test is based on the binomial distribution. Continuity correction was applied.

### Performance at a simulated higher-specificity operating point

When the performance of the AI system was evaluated at a predetermined higher-specificity operating point through simulations, the AI-assisted additional-reader workflow substantially reduced the proportion of cases requiring additional review to 2.4% (89/3,746), 3.0% (274/9,112) and 2.9% (457/15,953) for the initial pilot, extended pilot and live-use phases, respectively, while still detecting 5 of the 6 (1.3/1,000, a 10% relative increase) additional cancer cases found in the initial pilot, 11 of the 13 (1.2/1,000, a 9% relative increase) additional cancer cases found in the extended pilot and 10 of the 11 (0.6/1,000, a 4% relative increase) additional cancer cases found in live use (Table 4). Of the additional cancer cases, four (80.0%) in the initial pilot, nine (81.1%) in the extended pilot and five (50.0%) in live use were confirmed to be invasive; zero (0.0%) in the initial pilot, one (9.1%) in the extended pilot and two (20.0%) in live use were confirmed to be in situ cancer; and one (20.0%) in the initial pilot, one (9.1%) in the extended pilot and three (30.0%) in live use had missing invasiveness information.

**Table 4:** Outcome metrics for standard double reading versus double reading plus the AI-assisted additional-reader workflow at a higher-specificity operating point

Variable	Double reading		Double reading plus the AI-assisted additional-reader workflow		Difference
	Num/Denom	Value (95% CI)	Num/Denom	Value (95% CI)	
Results of phase 1, pilot rollout (1 site, 1 additional arbitrator, additional arbitration cases were single read), *n* = 3,746 screens					
CDR (per 1,000 cases)	48/3,746	12.8 (9.7–16.9)	53/3,746	14.1 (10.8–18.5)	1.3^a^
RR (%)	250/3,746	6.7 (5.9–7.5)	255/3,746	6.8 (6.0–7.7)	0.1
Sen (%)	48/57	82.8 (71.7–90.4)	53/57	93.0 (83.3–97.2)	8.8^a^
Spec (%)	3,487/3,689	94.5 (93.7–95.2)	3,487/3,689	94.5 (93.7–95.2)	0.0
PPV (%)	48/250	19.2 (14.8–24.5)	53/255	20.8 (16.3–26.2)	1.6
Arbitration rate (%)	114/3,746	3.0 (2.5–3.6)	203/3,746	5.4 (4.7–6.2)	2.4
Positive discordance rate (%)	–	–	89/3,746	2.4 (1.9–2.9)	–
RR of additional arbitration (%)	–	–	5/89	5.6 (2.4–12.5)	–
PPV of additional arbitration (%)	–	–	5/5	100 (56.6–100)	–
Results of phase 2, extended pilot (4 sites, 3 additional arbitrators, all additional arbitration cases were read by each additional arbitrator), *n* = 9,112 screens					
CDR (per 1,000 cases)	126/9,112	13.8 (11.6–16.4)	137/9,112	15.0 (12.7–17.7)	1.2^a^
RR (%)	639/9,112	7.0 (6.5–7.6)	653/9,112	7.2 (6.7–7.7)	0.2
Sen (%)	126/142	86.9 (80.4–91.4)	137/142	96.5 (92.0–98.5)	7.7^a^
Spec (%)	8,457/8,970	94.3 (93.8–94.7)	8,454/8,970	94.2 (93.7–94.7)	0.0
PPV (%)	126/639	19.7 (16.8–23.0)	137/653	21.0 (18.0–24.3)	1.3
Arbitration rate (%)	270/9,112	3.0 (2.6–3.3)	544/9,112	6.0 (5.5–6.5)	3.0
Positive discordance rate (%)	–	–	274/9,112	3.0 (2.7–3.4)	–
RR of additional arbitration (%)	–	–	14/274	5.1 (3.1–8.4)	–
PPV of additional arbitration (%)	–	–	11/14	78.6 (52.4–92.4)	–
Results of phase 3, live use in standard clinical practice (4 sites, 3 additional arbitrators, additional arbitration cases were single read), *n* = 15,953 screens					
CDR (per 1,000 cases)	238/15,953	14.9 (13.2–16.9)	248/15,953	15.5 (13.7–17.6)	0.6^a^
RR (%)	1,228/15,953	7.7 (7.3–8.1)	1,252/15,953	7.8 (7.4–8.3)	0.2
Sen (%)	238/251	94.1 (90.4–96.4)	248/251	98.8 (96.5–99.6)	4.0^a^
Spec (%)	14,712/15,702	93.7 (93.3–94.1)	14,698/15,702	93.6 (93.2–94.0)	−0.1
PPV (%)	238/1,228	19.4 (17.3–21.7)	248/1,252	19.8 (17.7–22.1)	0.4
Arbitration rate (%)	529/15,953	3.3 (3.0–3.6)	986/15,953	6.2 (5.8–6.6)	2.9
Positive discordance rate (%)	–	–	457/15,953	2.9 (2.6–3.1)	–
RR of additional arbitration (%)	–	–	24/457	5.3 (3.6–7.7)	–
PPV of additional arbitration (%)	–	–	10/24	41.7 (24.5–61.2)	–

See metric definitions in Methods.

^a^The two-sided McNemar test to assess CDR and Sen differences between double reading and double reading plus the AI-assisted additional-reader workflow resulted in *P* values of 0.063, 0.001 and <0.001 for phases 1, 2 and 3, respectively. The McNemar test is based on the binomial distribution. Continuity correction was applied.

## Discussion

This analysis of prospective real-world usage data provides evidence that using AI in clinical practice results in a measurable increase in breast cancer detection. We analyzed the effects of the AI-assisted additional-reader workflow in two pilot phases and found that the results were maintained when AI was used in daily screening practice. Moreover, the observed clinical benefit (a significant 5–13% increase in the rate of early detection of mostly invasive and small cancerous tumors) had minimal impact on recall rates, thereby demonstrating the possibility of increasing cancer detection with no false-positive additional recalls. Although the double-reading recall rate (6.7–7.7%) in this evaluation is in line with previous results published in the UK and Europe^9,15^, the double-reading CDR is higher (14/1,000) than previously reported^9^—possibly resulting from the resumption of breast cancer screening programs after the coronavirus disease pandemic. Nevertheless, the AI-assisted additional-reader workflow supported the screening service by further increasing the rate of early cancer detection. It also can potentially reduce the proportion of cases requiring additional arbitration review to <3% of cases while still achieving increased cancer detection by 0.5–1.3 per 1,000 cases, corresponding to a 4–10% relative increase in cancer detection using a higher-specificity operating point. Future work investigating the implementation of a variety of operating points would be needed to confirm the extent of achievable improvement in early cancer detection in the context of sites with different needs, capacities and screening population characteristics.

Implementing AI into the diagnostic workflow requires careful monitoring of continued performance over time^16^. For the AI-assisted additional-reader workflow, the effectiveness of downstream clinical assessments of recalled positive discordant cases should be examined to ensure that potential cancer cases are found. Moreover, the AI-assisted additional-reader workflow could be combined with workflows focused on workload savings, such as using AI as an independent second reader. Large-scale retrospective studies of the same AI system used in this assessment have demonstrated that AI as an independent second reader can offer up to 45% workload savings^8,9^, offsetting the 3–11% additional arbitration reads (1–6% additional overall reading workload) for the AI-assisted additional-reader workflow while providing the benefit of increased cancer detection.

The AI-assisted additional-reader workflow was designed to flag high-priority cases not recalled by standard double reading, likely making the flagged set of cases a more difficult or complex set to read. We believe that this would be helpful in the training of mammogram readers. The spectrum of disease detected with the AI-assisted additional-reader workflow will be assessed in future work covering features such as invasiveness, tumor size, grade and lymph node status.

Several limitations need to be considered when interpreting the presented results. First, data were collected from only one breast cancer screening institution (with four sites) in one country. As screening programs vary between clinical sites and countries, future studies must confirm the benefit of the AI-assisted additional-reader workflow in other settings and screening populations. Furthermore, as only one commercial AI system was evaluated, the results may not be representative of other commercially available systems. Additionally, given that the follow-up period in this prospective assessment ranged only from 2 to 9 months, no information is yet available about possible interval cancer cases in the studied population. A longer follow-up analysis is required for a more accurate assessment of AI’s potential for improving cancer detection in the context of interval cancer occurrence. Moreover, the impact of inter-reader variation on the AI-assisted additional-reader workflow’s screening outcomes remains unclear and needs to be assessed in follow-up work.

Despite the many challenges in developing, validating, deploying and monitoring AI to ensure patient safety, this evaluation shows that a commercially available AI system can be effectively deployed, with its previously predicted benefits realized in a prospective real-world assessment of a live clinical workflow. We believe that the findings highlight opportunities for using AI in breast screening while demonstrating concrete steps for its safe deployment. The phased prospective approach underlines the potential for various AI adoption pathways.

## Methods

### Datasets for analysis

This study is an analysis of postmarket data collected at MaMMa Klinika, a large breast cancer screening institution in Hungary. Structured query language was used to collect data. Custom code using Python software version 3.8.8 and open-source Python packages, including pandas version 1.2.4, NumPy version 1.20.1, sklearn version 0.24.1 and statsmodels version 0.12.2, were used for data analysis. The analysis complied with all relevant ethical regulations. External ethical review was not required as the AI system was used as part of the standard of care in the screening service at each implementation phase of this service evaluation. Ethical considerations were reviewed internally by the screening service provider, MaMMa Klinika. The evaluation used deidentified data and presented results in aggregate without listing data of individual screening participants to protect their anonymity. As a consequence, the evaluation also did not require patient consent.

### Metrics

Standard breast screening metrics, CDR and recall rate were primarily used to assess the effects of the AI-assisted additional-reader workflow compared to standard double reading without AI. CDR was calculated as the number of screen-detected cancer cases detected divided by the number of all screening cases. Recall rate was calculated as the number of cases recalled divided by the number of all cases; this should not be confused with the term ‘recall’ often used as a metric for sensitivity in machine learning. Arbitration rate was calculated as the number of arbitrations conducted divided by the number of all cases, with the double-reading arbitration rate including only double-reading arbitrations and the total arbitration rate including double-reading and additional-reader arbitrations. PPV was calculated as the number of screen-detected cancer cases divided by the number of recalled screens. Sensitivity was calculated as the number of screen-detected cancer cases divided by the number of all known positive screens. Specificity was calculated as the number of non-recalled screens divided by the number of all non-positive screens. Positive discordance rate was calculated as the number of AI-flagged positive discordant cases divided by the number of all cases. As the AI-assisted additional-reader workflow occurs subsequently to the double-reading workflow on the same cases, paired comparisons between the AI-assisted additional-reader and double-reading workflows were possible, with an exact measurement of the impact of AI in terms of additional recalls and cancer cases found. All detected cancer cases were confirmed with biopsy or histopathological examination within 12 months of the original screen or judged to be cancer by the patient tumor board (multidisciplinary team).

### Statistical analysis

No statistical method was used to predetermine sample sizes. No data were excluded from the analyses. Blinding was not required as randomization was not applied. The standard double-reading process did not involve the AI system, and readers were blinded to the AI system’s output during the double-reading process. The Wilson score method was used to calculate 95% CIs. The statistical significance of CDR differences was assessed using the McNemar test. A *P* value of <0.05 was defined as statistically significant.

### AI system

This evaluation used a commercially available AI system (Mia version 2.0, Kheiron Medical Technologies). The AI system is intended to process only cases from female participants and works with standard DICOM (Digital Imaging and Communications in Medicine) cases as inputs. The AI system analyzes four images with two standard full-field digital mammography views (craniocaudal and mediolateral oblique) per breast. The AI system’s primary output per case is a single binary recommendation of ‘recall’ (for further assessment based on findings suggestive of malignancy) or ‘no recall’ (no further assessment until the next screening interval). The AI system can provide binary recall recommendations for six predetermined operating points, ranging from having a balanced trade-off between sensitivity and specificity to having trade-offs that emphasize either sensitivity or specificity. The AI system’s balanced sensitivity/specificity and higher-specificity operating points are most relevant when the AI system is used in the AI-assisted additional-reader workflow. The set of cases flagged by the AI system’s higher-specificity operating point in the AI-assisted additional-reader workflow is always a subset of the cases flagged by the AI system’s balanced sensitivity/specificity operating point. Therefore, results at the higher-specificity operating point can be precisely simulated based on the balanced operating point results. The optionality between the different operating point trade-offs makes a significant difference for practical applicability at sites with differing workforces. Additionally, the AI system provides regions of interest indicating image locations showing characteristics most suggestive of malignancy. Depending on the clinical workflow and exact integration of the AI system, the AI’s recommendation may be used independently or combined with human reader assessment.

The underlying technology of the AI system is based on deep convolutional neural networks (CNNs), which are state-of-the-art machine learning tools for image classification. The AI system is a combination (also known as an ensemble) of multiple models with a diverse set of different CNN architectures. Each model was trained for malignancy detection. The final prediction of the ensemble is obtained by aggregating individual model outputs, with a subsequent threshold applied to the malignancy detection score to generate a binary recommendation of ‘recall’ or ‘no recall’. The thresholds relate to one of the AI system’s six predetermined, clinically meaningful operating points according to desired sensitivity/specificity trade-offs.

The AI system was trained on a heterogeneous, large-scale collection of more than 1 million images from real-world screening programs across different countries, multiple sites and equipment from different vendors over a period of >10 years. Positive cases were defined as pathology-proven malignancies confirmed by fine-needle aspiration cytology, core needle biopsy, vacuum-assisted core biopsy and/or histological analysis of surgical specimens. Negative cases were confirmed through multiple years of follow-up.

The AI software version and operating points used in the present evaluation were fixed before each phase. None of the evaluation data were used in any aspect of algorithm development.

The AI system’s performance, generalizability and clinical utility were previously confirmed in a large-scale retrospective AI generalizability study^8,9,14^. The study demonstrated that double reading with the AI system, compared to human double reading, resulted in at least noninferior recall rate, CDR, sensitivity, specificity and PPV for each mammography vendor and site, with superior recall rate, specificity and PPV observed for some mammography vendors and sites^9^. The double-reading simulation with the AI system indicated that using AI as an independent reader (in all cases it could process) can result in a 3.3–12.3% increase in the arbitration rate^9^ but can reduce human workload by 30.0–44.8%. AI as a supporting reader (used as a second reader only when it agrees with the first human reader) was found to be superior or noninferior on all screening metrics compared to human double reading while nearly halving the number of arbitrations (from 3.4% to 1.8%) and reducing the number of cases requiring second human reading (by up to 87%)^8^. Additionally, no differences in prognostic features (invasiveness, grade, tumor size and lymph node status) were found between the cancer cases detected by the AI system and those detected by human readers^14^. These findings imply that cancer cases detected by the AI system and human readers are likely to have similar clinical courses and outcomes, with limited or no downstream effects on screening programs, supporting the potential role of AI as a reader in the double-reading workflow.

### Reporting summary

Further information on research design is available in the Nature Portfolio Reporting Summary linked to this article.

## Online content

Any methods, additional references, Nature Portfolio reporting summaries, source data, extended data, supplementary information, acknowledgements, peer review information; details of author contributions and competing interests; and statements of data and code availability are available at 10.1038/s41591-023-02625-9.

### Supplementary information

Reporting Summary

## Data Availability

Access to patient-level data and supporting clinical information can be made available upon request, contingent on patient privacy and confidentiality obligations and subject to information governance at MaMMa Klinika (Hungary). Data access requests can be made to the corresponding author by email at annie@kheironmed.com and will be processed within 4 weeks.

## Code availability

The code used for training and deploying the evaluated AI system has many dependencies on internal tooling, proprietary components, infrastructure and hardware. Therefore, full code release is not feasible. We provide a technical description of the AI system in the online Methods, together with a code repository to facilitate the reproducibility of research involving deep learning models for breast cancer detection using digital mammography. The code provided at https://github.com/Kheiron-Medical/mammo-net demonstrates the training and testing of state-of-the-art CNNs that build the core component of most commercially available AI systems for breast cancer detection.
