# Supplementary material for: Prospective implementation of AI-assisted screen reading to improve early detection of breast cancer
Source: Nat Med. 2023 Nov 16;29(12):3044–9. doi: 10.1038/s41591-023-02625-9 (PMC10719086; doi:10.1038/s41591-023-02625-9)
Supplement: Supplementary file 1 — Reporting Summary [file 41591_2023_2625_MOESM1_ESM.pdf]

nature portfolio

Corresponding author(s): Dr. Annie Ng

Last updated by author(s): Oct 3, 2023

Reporting Summary

Nature Portfolio wishes to improve the reproducibility of the work that we publish. This form provides structure for consistency and transparency in reporting. For further information on Nature Portfolio policies, see our Editorial Policies and the Editorial Policy Checklist.

Statistics

For all statistical analyses, confirm that the following items are present in the figure legend, table legend, main text, or Methods section.

n/a	Confirmed	
☐	☒	The exact sample size (*n*) for each experimental group/condition, given as a discrete number and unit of measurement
☐	☒	A statement on whether measurements were taken from distinct samples or whether the same sample was measured repeatedly
☐	☒	The statistical test(s) used AND whether they are one- or two-sided<br>*Only common tests should be described solely by name; describe more complex techniques in the Methods section.*
☒	☐	A description of all covariates tested
☒	☐	A description of any assumptions or corrections, such as tests of normality and adjustment for multiple comparisons
☐	☒	A full description of the statistical parameters including central tendency (e.g. means) or other basic estimates (e.g. regression coefficient) AND variation (e.g. standard deviation) or associated estimates of uncertainty (e.g. confidence intervals)
☐	☒	For null hypothesis testing, the test statistic (e.g. *F*, *t*, *r*) with confidence intervals, effect sizes, degrees of freedom and *P* value noted<br>*Give P values as exact values whenever suitable.*
☒	☐	For Bayesian analysis, information on the choice of priors and Markov chain Monte Carlo settings
☒	☐	For hierarchical and complex designs, identification of the appropriate level for tests and full reporting of outcomes
☒	☐	Estimates of effect sizes (e.g. Cohen's *d*, Pearson's *r*), indicating how they were calculated

Our web collection on statistics for biologists contains articles on many of the points above.

Software and code

Policy information about availability of computer code

Data collection	SQL was used to collect the data.
Data analysis	Custom code using Python software Version 3.8.8 and open source Python packages including Pandas version 1.2.4, Numpy version 1.20.1, Sklearn version 0.24.1, and Statsmodels version 0.12.2 have been used to analyse the data.<br><br>The code used for training and deploying the evaluated AI system has a large number of dependencies on internal tooling, proprietary components, infrastructure and hardware. The full code release is therefore not feasible. We provide a technical description of the AI system in the online Methods section together with a code repository to facilitate reproducibility of research involving deep learning models for breast cancer detection in digital mammography. The code provided under https://github.com/Kheiron-Medical/mammo-net demonstrates the training and testing of state-of-the-art convolutional neural networks which build the core component of most commercially available breast cancer AI systems.

For manuscripts utilizing custom algorithms or software that are central to the research but not yet described in published literature, software must be made available to editors and reviewers. We strongly encourage code deposition in a community repository (e.g. GitHub). See the Nature Portfolio guidelines for submitting code & software for further information.

Data

Policy information about availability of data

All manuscripts must include a data availability statement. This statement should provide the following information, where applicable:

- Accession codes, unique identifiers, or web links for publicly available datasets
- A description of any restrictions on data availability
- For clinical datasets or third party data, please ensure that the statement adheres to our policy

Subject to patient privacy and confidentiality obligations, access to patient-level data and supporting clinical information can be made available upon request and subject to information governance at MaMMa Klinika (Hungary). Data access requests will be processed within four weeks. Such requests can be made to the corresponding author by email at annie@kheironmed.com.

Research involving human participants, their data, or biological material

Policy information about studies with human participants or human data. See also policy information about sex, gender (identity/presentation), and sexual orientation and race, ethnicity and racism.

Reporting on sex and gender	Only female participants have been included in any analysis to represent the AI system's intended use population, which also reflects breast cancer screening practice.
Reporting on race, ethnicity, or other socially relevant groupings	Race, ethnicity, and other social groupings were not reported.
Population characteristics	The initial pilot included 3,746 women with an average age of 58.2 years (standard deviation (SD) 11.0). Of these, 126 (3.4%) had a family history of cancer and 479 (12.7%) had a Tabar parenchymal pattern classification of 4 or 5, indicating high density. In the extended pilot (N=9,112), the mean age was also 58.2 years (SD 10.7). Tabar classification of 4 or 5 was identified for 1,094 women (12.0%) and 274 (3.0%) had a family history of cancer. Finally, in the live use phase, screens of 15,953 women were included. The mean age was 58.6 years (SD 10.5), 615 (3.9%) women had a family history of cancer and 1,733 (10.8%) of the cohort had a Tabar classification of 4 or 5.
Recruitment	All screening participants whose case was double read by human double reading and could be processed by the AI system were included in any analyses.
Ethics oversight	External ethical review was not required as the AI system was used as a part of standard of care in a service evaluation at the screening service at each implementation phase. Ethical considerations were reviewed internally by the screening service provider, MaMMa Klinika. The evaluation used data that was de-identified and presented results in aggregate, never listing individual screening participant data, to protect the anonymity of individual screening participants. The evaluation also did not require patient consent as a consequence.

Note that full information on the approval of the study protocol must also be provided in the manuscript.

Field-specific reporting

Please select the one below that is the best fit for your research. If you are not sure, read the appropriate sections before making your selection.

☒ Life sciences ☐ Behavioural & social sciences ☐ Ecological, evolutionary & environmental sciences

For a reference copy of the document with all sections, see nature.com/documents/nr-reporting-summary-flat.pdf

Life sciences study design

All studies must disclose on these points even when the disclosure is negative.

Sample size	No formal sample sizing was conducted. The length of the first and second phases of work presented was based on the site's workload capacity, and the third phase of live use is ongoing in practice, but results are presented through January 2023 to enable sufficient follow-up for the collection of positive information.
Data exclusions	Only data that included double reading opinions that the AI could process were included. The AI is not intended to process cases from non-female participants and cases that do not exactly include the standard 4-view screening images.
Replication	The analysis was repeated and cross checked internally by two separate analysts to ensure correctness. The results were successfully replicated.
Randomization	All eligible participants from defined time frames and sites were included in the analyses, thus randomization was not conducted or required.
Blinding	Reading practices were carried out according to normal standard practice.The standard double reading process did not involve the AI system,

Blinding	and readers were blinded to the AI system's output during the double reading process. No further blinding procedures were implemented to ensure results were representative of real clinical practice.

Reporting for specific materials, systems and methods

We require information from authors about some types of materials, experimental systems and methods used in many studies. Here, indicate whether each material, system or method listed is relevant to your study. If you are not sure if a list item applies to your research, read the appropriate section before selecting a response.

Materials & experimental systems

n/a	Involved in the study
☒	☐ Antibodies
☒	☐ Eukaryotic cell lines
☒	☐ Palaeontology and archaeology
☒	☐ Animals and other organisms
☒	☐ Clinical data
☒	☐ Dual use research of concern
☒	☐ Plants

Methods

n/a	Involved in the study
☒	☐ ChIP-seq
☒	☐ Flow cytometry
☒	☐ MRI-based neuroimaging
